# The predictive value of 53BP1 and BRCA1 mRNA expression in advanced non-small-cell lung cancer patients treated with first-line platinum-based chemotherapy

**DOI:** 10.18632/oncotarget.1157

**Published:** 2013-07-31

**Authors:** Laura Bonanno, Carlota Costa, Margarita Majem, Jose Javier Sanchez, Ana Gimenez-Capitan, Ignacio Rodriguez, Alain Vergenegre, Bartomeu Massuti, Adolfo Favaretto, Massimo Rugge, Cinta Pallares, Miquel Taron, Rafael Rosell

**Affiliations:** ^1^ Second Medical Oncology Unit, Istituto Oncologico Veneto I.R.C.C.S, Via Gattamelata 64, Padova, Italia; ^2^ Pangaea Biotech S.L, USP Dexeus University Institute, Barcelona, Spain; ^3^ Hospital de Sant Pau, Barcelona, Spain; ^4^ Universidad Autonoma de Madrid, Madrid, Spain; ^5^ Unidad Epidemiología y Estadística, Departamento de Obstetricia, Ginecología y Reproducción, USP Dexeus University Institute, Barcelona, Spain; ^6^ Cluzeau Hospital, Limoges, France; ^7^ Hospital General de Alicante, Alicante, Spain; ^8^ Department of Medicine, Surgical Pathology and Cytopathology Unit, Università degli Studi di Padova, Padova, Italy; ^9^ Catalan Institute of Oncology, Hospital Germans Trias i Pujol, Badalona, Barcelona, Spain

**Keywords:** BRCA1, 53BP1, DNA repair, predictive modeling, platinum

## Abstract

Platinum-based chemotherapy is the standard first-line treatment for non-oncogene-addicted non-small cell lung cancers (NSCLCs) and the analysis of multiple DNA repair genes could improve current models for predicting chemosensitivity. We investigated the potential predictive role of components of the 53BP1 pathway in conjunction with *BRCA1*. The mRNA expression of *BRCA1*, *MDC1*, *CASPASE3*, *UBC13*, *RNF8*, *53BP1*, *PIAS4*, *UBC9* and *MMSET* was analyzed by real-time PCR in 115 advanced NSCLC patients treated with first-line platinum-based chemotherapy. Patients expressing low levels of both *BRCA1* and *53BP1* obtained a median progression-free survival of 10.3 months and overall survival of 19.3 months, while among those with low *BRCA1* and high *53BP1* progression-free survival was 5.9 months (P <0.0001) and overall survival was 8.2 months (P=0.001). The expression of *53BP1* refines BRCA1-based predictive modeling to identify patients most likely to benefit from platinum-based chemotherapy.

## INTRODUCTION

The standard first-line treatment of advanced non-small-cell lung cancer (NSCLC) in patients with wild-type epidermal growth factor receptor (EGFR) is platinum-based chemotherapy. However, median overall survival (OS) remains less than 12 months, with great inter-individual variability in efficacy and toxicity. Platinums act mainly by damaging DNA, and DNA repair capacity is thus a mechanism of resistance. Molecular predictive markers of sensitivity to platinum-based chemotherapy are needed to optimize the therapeutic potential of chemotherapy in this disease setting. In addition, the therapeutic window of the drug could be improved by investigating molecular predictive markers of specific toxicity, opening new therapeutic perspectives such as the association of inhibitors of PKCσ [1].

The protein BRCA1 plays an important role in the repair of bulky DNA adducts by nucleotide excision repair (NER), mainly in the sub-pathway repairing the damage on actively transcribed DNA (transcription-coupled NER) [2, 3]. BRCA1 is also a main component of DNA double-strand break repair through the error-free mechanism of homologous recombination [4]. The pivotal role of BRCA1 in double-strand break repair may be modulated by interaction with other components of homologous recombination [5]. In preclinical models, BRCA1 expression conferred resistance to cisplatin and sensitivity to taxanes [6-10], and its predictive role has been confirmed in several solid tumors, including NSCLC [11-15]. In particular, the results of a prospective phase II trial demonstrated the feasibility of assessing *BRCA1* mRNA expression by real-time PCR in the clinical setting, and additional retrospective analyses indicated that other DNA repair components could modulate the BRCA1 predictive model. The retrospective analysis of patients enrolled in the trial showed that mRNA expression levels of receptor associated protein 80 (*RAP80*), which is involved in the recruitment of BRCA1 to DNA damage sites, was able to refine the BRCA1-based predictive model in patients expressing low levels of *BRCA1* treated with platinum-based chemotherapy [16].

**Figure 1:**
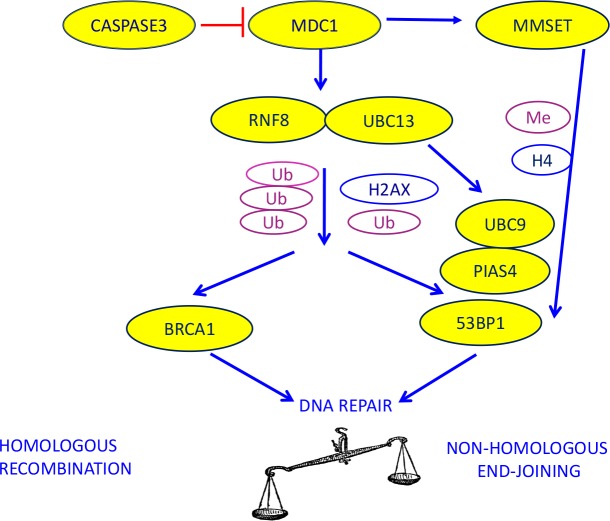
The biological model providing the rationale for our choice of DNA repair components to evaluate as potential predictive markers in advanced NSCLC patients treated with platinum-based chemotherapy

The protein BRCA1 is recruited at the sites of DNA damage through a mechanism that includes the localization of the MRE11-RAD50-NBS1 (MRN) complex at double-strand break sites, the activation of phosphoinositide-3-kinase-like kinases (PIKKs), including ATM and DNA-PK, and the phosphorylation of histone H2AX proteins [17]. This process leads to the binding of mediator of DNA damage checkpoint 1 (MDC1), which initiates the assembly of the DNA repair complexes. The protein MDC1 is also a target for specific cleavage by CASPASE 3, a major component of the apoptotic pathway. This specific cleavage between the forkhead-associated (FHA) and breast cancer C-terminal (BRCT) domains can prevent the activation of DNA damage repair [18]. MDC1 also recruits the UBC13-RNF8 complex, which facilitates the accumulation of BRCA1 at damaged DNA through post-translational protein modification (ubiquitination) [17, 19, 20] (Figure 1).

MDC1, the initiator of DNA damage response to double-strand breaks, can be cleaved by CASPASE3, thus preventing DNA repair and enhancing apoptosis. MDC1 recruits the mediators of DNA repair through ubiquitination, in which RNF8 and UBC13 are involved. This leads to the assembly and the activation of both the BRCA1 and the 53BP1 pathways. 53BP1 can also be induced by MMSET activation and needs post-translational modifications induced by PIAS4 and UBC9.

The 53 binding protein 1 (53BP1) plays an important role in modulating BRCA1-driven DNA damage response [21, 22]. 53BP1 was originally identified as being able to bind to wild-type – but not to mutant – p53 [23]. Later, preclinical data showed that 53BP1 is also able to mediate double-strand break repair, particularly through error-prone non-homologous end-joining (NHEJ) [24]. 53BP1 modulates the chromatin structure at DNA damage sites and contributes to maintaining genomic stability [25]. In addition, it negatively regulates homologous recombination repair by inhibiting CTIP [22], which creates a complex with BRCA1 to promote homologous recombination. While 53BP1 has been found to localize at both endogenous and exogenous double-strand breaks in a cell-cycle dependent manner, the phosphorylated forms have been detected only in response to exogenous double-strand breaks generated by ionizing radiation and mediated by ATM and DNA-PK [26].

The 53BP1 pathway is activated after the recruitment of RNF8-UBC13 by MDC1, but it can also be mediated by methyltransferase multiple myeloma SET domain (MMSET), which is overexpressed in several solid tumors [27, 28] (Figure 1). The function of 53BP1 in DNA repair is also positively modulated by sumoylation, a post-translational protein modification induced by PIAS4 and UBC9 [29] (Figure 1).

However, to the best of our knowledge, the potential predictive role of the 53BP1 pathway has not yet been examined in advanced NSCLC in the clinical setting.

In order to shed light on the potential influence of components of the 53BP1 pathway on the BRCA1 predictive model, we retrospectively analyzed the expression levels of *BRCA1*, *MDC1*, *CASPASE3*, *RNF8*, *UBC13*, *53BP1*, *PIAS4*, *UBC9* and *MMSET* (Figure 1) in tumors from advanced NSCLC patients and correlated our results with outcome to first-line platinum-based chemotherapy.

## RESULTS

### Clinical outcome

**Figure 2:**
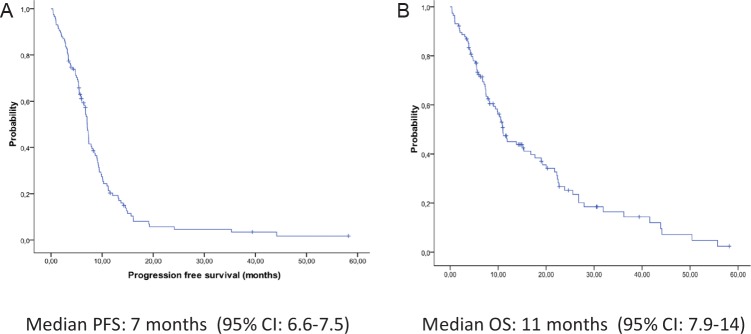
Kaplan-Meier curves of (A) progression-free survival and (B) overall survival for all 115 patients

The median PFS of the overall study population (115 patients) was 7 months (95% CI, 6.6-7.5) and the median OS was 11 months (95% CI, 7.9-14) for all 115 patients (Figure 2). Radiological response was assessed in 102 patients (89%). The overall response rate was 35%, including 3 complete radiological responses, and 37% of patients had stable disease as the best radiological response (Table 1). Performance status (PS) of 0-1 and female gender were clinical markers of better prognosis. PFS and OS for the 83 patients with PS 0-1 were 7.4 (95% CI, 6-8.8) and 12 (95% CI, 5.4-18. 4) months, respectively, compared to 3 (95% CI, 1-2.5) and 3.8 (95% CI, 2.5-5) months, respectively, for the 26 patients with PS 2 (P<0.001) (Supplementary Appendix, Figure S1). Female patients had longer PFS and OS than males (P=0.003) (data not shown). No association was observed between smoking status or chemotherapy regimen and outcome (data not shown).

**Table 1:** Patient characteristics of 115 advanced NSCLC patients treated with platinum-based chemotherapy

	N (%)
Age median (range)	64 (40-82)
Gender Male Female	87 (76) 28 (24)
ECOG performance status 0 1 2 Not available	13 (11) 70 (61) 26 (23) 6 (5)
Smoking status Current smoker Former smoker Never smoker	55 (48) 46 (40) 14 (12)
Stage IIIB IV	7 (6) 108 (94)
Histology Adenocarcinoma Squamous cell carcinoma Large cell carcinoma Adenosquamous carcinoma	67 (58) 26 (23) 21 (18) 1 (1)
First-line treatment Cisplatin-gemcitabine Carboplatin-gemcitabine Cisplatin-pemetrexed Carboplatin-pemetrexed	51 (44) 36 (31) 14 (12) 14 (12)
Second-line treatment Taxanes EGFR TKIs Others	13 (11) 24 (21) 14 (12)
Response rate Complete response Partial response Stable disease Progressive disease Not recorded	3 (3) 37 (32) 43 (37) 19 (17) 13 (11)

Six (5%) tumor samples harbored *EGFR* mutations: four exon 19 deletions and two L858R mutations in exon 21. The presence of *EGFR* mutations was not associated with outcome. At the time of data collection, one of the six patients had received erlotinib as second-line treatment for more than one month and another had received erlotinib as second-line treatment for three weeks.

### Gene expression

Adequate quality and quantity RNA was extracted in 101 cases (88%). The number of cases in which gene mRNA expression was successfully analyzed varied for each gene (Supplementary Appendix, Table S2). High levels of correlation were observed between the mRNA expression of *53BP1* and the genes directly involved in the activation of its pathway: *UBC9* (ρ: 0.6, P<0.001) and *PIAS4* (ρ: 0.67, P<0.001), while low levels of correlation were observed between *BRCA1* and *53BP1* (ρ:0.37, P=0.003) (Supplementary Appendix, Table S3).

**Figure 3:**
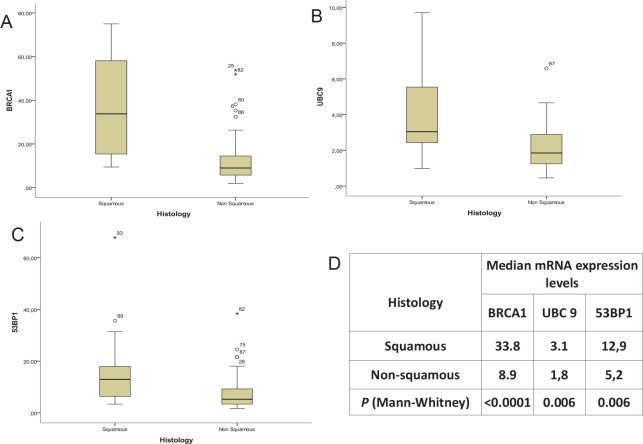
The expression levels of (A) BRCA1, (B) UBC9 and (C) 53BP1 in squamous versus non-squamous histology, with (D) median expression levels and P-values

The mRNA expression levels of *BRCA1*, *53BP1* and *UBC9* were significantly higher in tumors with squamous than in those with non-squamous histology (Figure 3). This difference was especially striking for *BRCA1*, where mRNA levels (expressed as ∆∆CT) were 33.8 for squamous histology and 8.9 for non-squamous (P<0.0001) (Figure 3). The mRNA expression of *BRCA1* also correlated with smoking status; non-smokers had significantly lower levels of *BRCA1* mRNA (5.5 vs 13; P=0.04) (Supplementary Appendix, Figure S2).

No association was observed between the mRNA expression of any of the individual genes and PFS or OS.

### Integrated gene expression analysis: the BRCA1-53BP1 predictive model

Based on the rationale of the biological model shown in Figure 1 and on preclinical data showing a potential influence of 53BP1 on the sensitivity of BRCA1-depleted cells to DNA-damaging agents and on their capacity for homologous recombination repair [22, 30], we then examined the effect on PFS and OS of the mRNA expression levels of both *BRCA1* and *53BP1* in combination. In particular, we hypothesized that *53BP1* could affect the platinum sensitivity of tumors already classified according to their levels of *BRCA1* mRNA expression. We, therefore, examined the effect of *53BP1* expression levels on the outcome of patients expressing low levels of *BRCA1* and, separately, on that of patients expressing high levels of *BRCA1*. The mRNA expression of *53BP1* mRNA was successfully analyzed in 74 cases, and *BRCA1* expression was successfully analyzed in 67 (Supplementary Appendix, Table S2). Expression levels of both genes were available in 62 cases.

**Figure 4:**
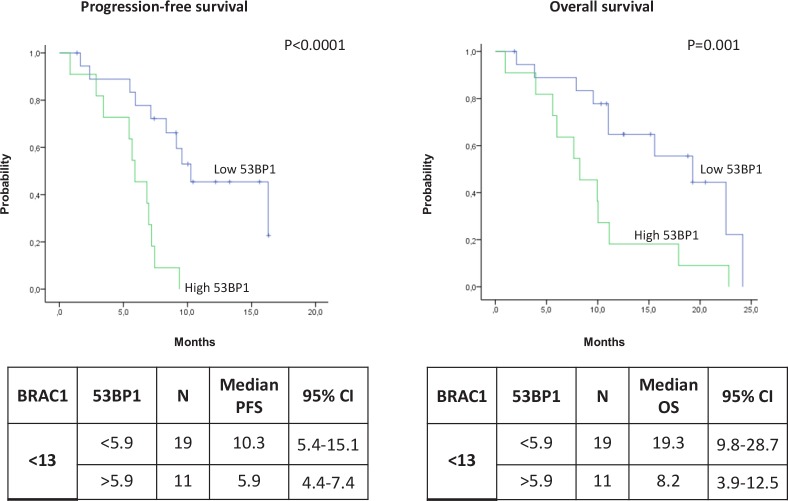
Kaplan-Meier curves showing (A) progression-free survival and (B) overall survival in patients with low BRCA1 expression according to 53BP1 expression levels

**Figure 5:**
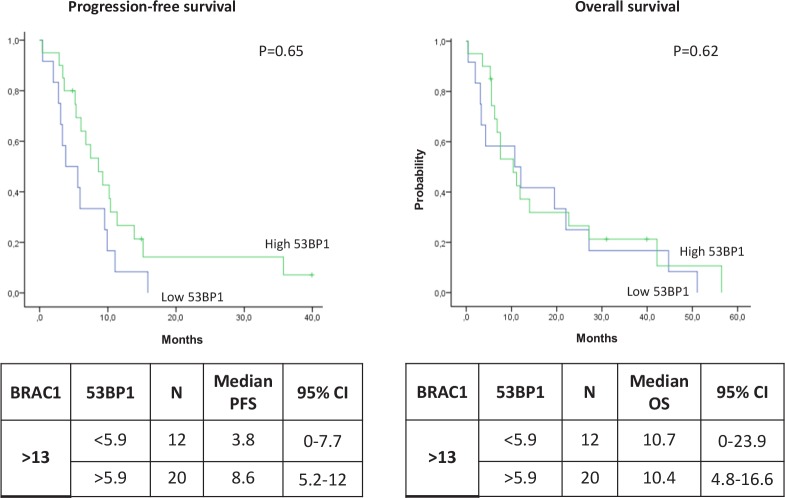
Kaplan-Meier curves showing (A) progression-free survival and (B) overall survival in patients with high BRCA1 expression according to 53BP1 expression levels

Among the patients expressing low levels of *BRCA1*, the median PFS was 10.3 months (95% CI, 5.4-15.1) for patients with low levels of *53BP1* and 5.9 months (95% CI, 4.4-7.4) for those with high 53BP1 levels (P<0.0001) (Figure 4A). The median OS was 19.3 months (95% CI, 9.8-28.7) in the presence of low levels of *53BP1* but decreased to 8.2 (95% CI, 3.9-12.5) when *53BP1* levels were high (P=0.001) (Figure 4B). In contrast, among patients with high levels of *BRCA1*, the *53BP1* mRNA expression did not affect the outcome. The median PFS was 8.6 months (95% CI, 5.2-12) for patients with high *53BP1* expression and 3.8 months (95% CI, 0-7.7) for those with low 53BP1 expression (P=0.65) (Figure 5A). OS was approximately 10 months in both sub-groups (P=0.62) (Figure 5B).

Overall disease control (including complete response, partial response and stable disease) was 75% in the group of patients with low levels of both *BRCA1* and *53BP1*, compared to 60% in patients with low *BRCA1* and high *53BP1* expression.

## DISCUSSION

While preclinical findings and retrospective studies have indicated that BRCA1 is a differential modulator of outcome to taxane- and platinum-based chemotherapy [7, 8, 10, 11, 31], to date no results are available to support the routine clinical use of BRCA1 expression as a predictive marker. At the same time, increasing knowledge concerning DNA repair pathways suggests the potential predictive role of additional DNA repair components that modulate BRCA1 function. We have examined the potential predictive role of the mRNA expression of several genes involved in the 53BP1 pathway.

We observed a different expression pattern according to histology, with higher levels of *BRCA1*, *53BP1* and *UBC9* expression in squamous cell carcinomas. These results are consistent with recently published data on a panel of potential predictive markers of platinum sensitivity in resected NSCLC patients, where some DNA repair genes had predictive value only in squamous cell carcinomas [32]. However, histology did not affect patient outcome in the present study, suggesting that perhaps future analyses should be performed separately in squamous and non-squamous histologies. The low number of squamous cell carcinomas in our study precluded a sub-analysis of patients according to histology, and this issue warrants further investigation.

We found no association between any of the individual genes and outcome to platinum-based chemotherapy, which reflects the complexity of the biological model at the center of the study (Figure 1). However, we have identified a novel two-gene predictive model for platinum-treated advanced NSCLC, based on the mRNA expression of *BRCA1* and *53BP1*. The patients who benefited most from first-line platinum-based treatment were those expressing low levels of both *BRCA1* and *53BP1*, who attained an impressive median PFS of 10.3 months and OS of 19.3 months. Importantly, these outcomes were attained in spite of the inclusion of PS 2 patients, which indicates the strength of the predictive model.

The proteins BRCA1 and 53BP1 are recruited at DNA damage sites by mainly overlapping mechanisms, and it seems plausible that the two pathways interact, modulating the response to double-strand breaks and creating a balance between error-free homologous recombination repair and error-prone NHEJ [22] (Figure 1). The 53BP1 nuclear foci formation is specifically induced by double-strand breaks [33], and a direct interaction between BRCA1 and 53BP1, the two protagonists of double-strand break repair, has been demonstrated in preclinical models, where 53BP1 has been shown to be a positive transcriptional regulator of the BRCA1 promoter [34, 35]. In the experimental setting, the loss of 53BP1 reduces genomic instability and could partially restore the homologous recombination capacity of cells in the absence of BRCA1 [21, 22]. Nevertheless, experimental models of cells depleted of both BRCA1 and 53BP1 showed high levels of sensitivity to cisplatin and other agents – greater than that of cells depleted of only BRCA1 [30] – even though the cells demonstrated a similar capacity for homologous recombination to that of cells expressing the two genes [22].

**Figure 6:**
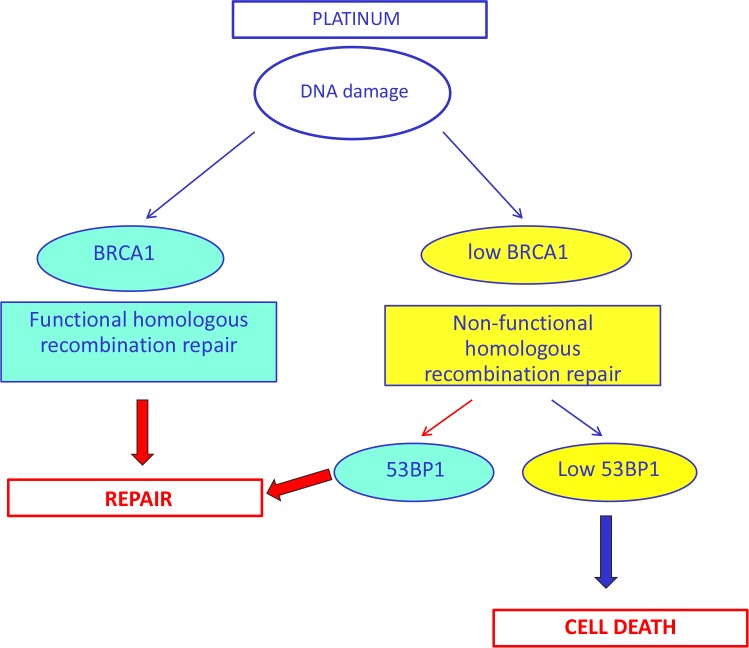
Simplified hypothetical model of the potential interplay between BRCA1 and 53BP1 in DNA damage response, based on our clinical results

In the present study, we have demonstrated in the clinical setting that low levels of *53BP1* mRNA are essential to maintain cisplatin sensitivity in the presence of low *BRCA1* levels, which is consistent with the biological model (Figure 1). This suggests that in the absence of BRCA1, the repair of interstrand crosslinks can occur in a homologous recombination-independent manner, which could be affected by 53BP1 (Figure 6). In contrast, in patients expressing high levels of *BRCA1*, *53BP1* expression did not affect outcome to platinum-based chemotherapy. We can speculate that in the presence of high *BRCA1* expression, which confers high capacity for homologous recombination – and consequently, greater tumor resistance to platinum therapy – 53BP1 might not be essential for determining platinum sensitivity.

The lack of homologous recombination capacity could be compensated for by the presence of 53BP1, leading to DNA repair in a homologous recombination-independent manner.

In addition to its predictive value, the model suggested by the present study could represent a novel model of synthetic lethality and could be exploited from the therapeutic point of view as is the case with BRCA1-2 mutated tumors treated with Poly ADP-ribose polymerase (PARP) inhibitors. In this case, the inhibition of PARP leads to the accumulation of single-strand breaks that are converted to double-strand breaks through the stalling and collapse of replication forks. BRCA1-2 mutations cause deficient homologous recombination and consequent unrepaired double-strand breaks, which cause cell death [36, 37]. This deficient homologous recombination sensitizes tumor cells to PARP inhibition. Interestingly, the synthetic lethality attained with PARP inhibition may also be driven by molecular alterations in genes involved in homologous recombination, including decreased BRCA1 mRNA expression [38]. In particular, aberrations of the Mre11-Rad50-Nbs1 (MRN) complex sensitized cells to PARP inhibition, even in BRCA1-2 wild-type tumors [39]. The potential therapeutic applications of synthetic lethality models could involve the clinical development of PARP inhibitors in the setting of molecularly-driven disease as well as the use of molecules inhibiting specific BRCA1 protein domains [5].

The present study has some limitations, including its retrospective nature and the relatively small number of patients with mRNA expression data available for both *BRCA1* and *53BP1*. Nevertheless, the highly significant results and the great difference in the absolute values of PFS and OS according to mRNA levels of *53BP1* among patients with low *BRCA1* expression show a clear predictive potential for this novel two-gene model. Our model warrants validation in a larger patient cohort, and the influence of other biomarkers of sensitivity to gemcitabine and pemetrexed should also be taken into account.

In conclusion, to the best of our knowledge, this is the first study to examine the potential predictive role in lung cancer of a series of DNA repair genes involved in the 53BP1 pathway. While *BRCA1* expression in isolation was not able to predict sensitivity to platinum-based chemotherapy in advanced NSCLC, we have identified a novel predictive two-gene model based on the impact of *53BP1* on *BRCA1* function. These findings confirm biological data on the complex interplay between BRCA1 and 53BP1 and the effect of 53BP1 in cells with low homologous recombination capacity. They pave the way for future studies of *53BP1* and other genetic events in NSCLC that may modulate the gene landscape imposed by *BRCA1*.

## METHODS

### Study population

Tumor samples from 115 patients with advanced NSCLC were collected retrospectively. Fifty-two patients were from the Istituto Oncologico Veneto (Padova, Italy), 51 from the Hospital Sant Pau (Barcelona, Spain), seven from the Hospital du Cluzeau (Limoges, France), and five from the Hospital General de Alicante (Alicante, Spain). Table 1 shows patient clinical characteristics.

The main inclusion criteria were: stage IIIB or IV NSCLC (sixth TNM staging system); first-line treatment with carboplatin or cisplatin plus gemcitabine or pemetrexed; no previous chemotherapy or radiotherapy; available tumor tissue and clinical data. Informed consent was obtained from all patients or their guardians. All the samples were screened for the presence of *EGFR* mutations.

### Gene expression analysis

The formalin-fixed paraffin-embedded specimens were stained with haematoxylin and eosin and evaluated by the pathologist of the Pangaea Biotech Molecular Biology Laboratory at USP Dexeus University Institute (Barcelona, Spain). Samples with more than 90% of tumor cells were processed using macrodissection; in those with less than 90%, laser microdissection was performed as previously described [40]. All samples used for RNA extraction had less than 10% of lymphocytes, necrosis or stromal cells. After deparaffinization and lysation, RNA extraction, retrotranscription and real-time PCR were performed as previously described [40]. Primers and probes for gene expression analysis of *BRCA1*, *MDC1*, *CASPASE3*, *RNF8*, *UBC13*, *53BP1*, *PIAS4*, *UBC9* and *MMSET* (Supplementary Appendix, Table S1) were designed according to their reference sequence in http://www.ncbi.nlm.nih.gov/sites/entrez?db=gene and the criteria of Applied Biosystems (Foster City, CA). The mRNA levels were measured according to the comparative Ct method, using β-actin as endogenous control and commercial RNA controls as calibrators.

### Statistical analyses

Gene expression levels were considered as categorical variables using the median value as cut-off point. Progression-free survival (PFS) was calculated from the beginning of treatment until radiological or clinical progression or death from any cause. OS was calculated from the beginning of treatment to death from any cause. Radiological response was assessed according to the Response Evaluation Criteria in Solid Tumors (RECIST) version 1.0. Median PFS and OS were estimated with the Kaplan-Meier method and compared with a two-sided log-rank test. The Mann-Whitney test was used for continuous variables and the Chi-Square or Fisher exact test for categorical variables. Spearman's correlation coefficient analysis was used to determine the correlation among different genes. The association between each potential prognostic factor and PFS or OS was assessed with a univariate Cox regression analysis. All analyses were performed using Statistical Package for Social Science (SPSS) for Windows version 17 (Chicago, IL). Significance was set at P≤0.05.

## Supplementary Figures and Tables




