# Supplementary material for: The predictive value of 53BP1 and BRCA1 mRNA expression in advanced non-small-cell lung cancer patients treated with first-line platinum-based chemotherapy
Source: Oncotarget. 2013 Jul 31;4(10):1572–81. doi: 10.18632/oncotarget.1157 (PMC3858546; doi:10.18632/oncotarget.1157)
Supplement: Supplementary file 1 [file oncotarget-04-1572-s001.pdf]

The predictive value of 53BP1 and BRCA1 mRNA expression in advanced non-small-cell lung cancer patients treated with first-line platinum-based chemotherapy - Bonanno et al

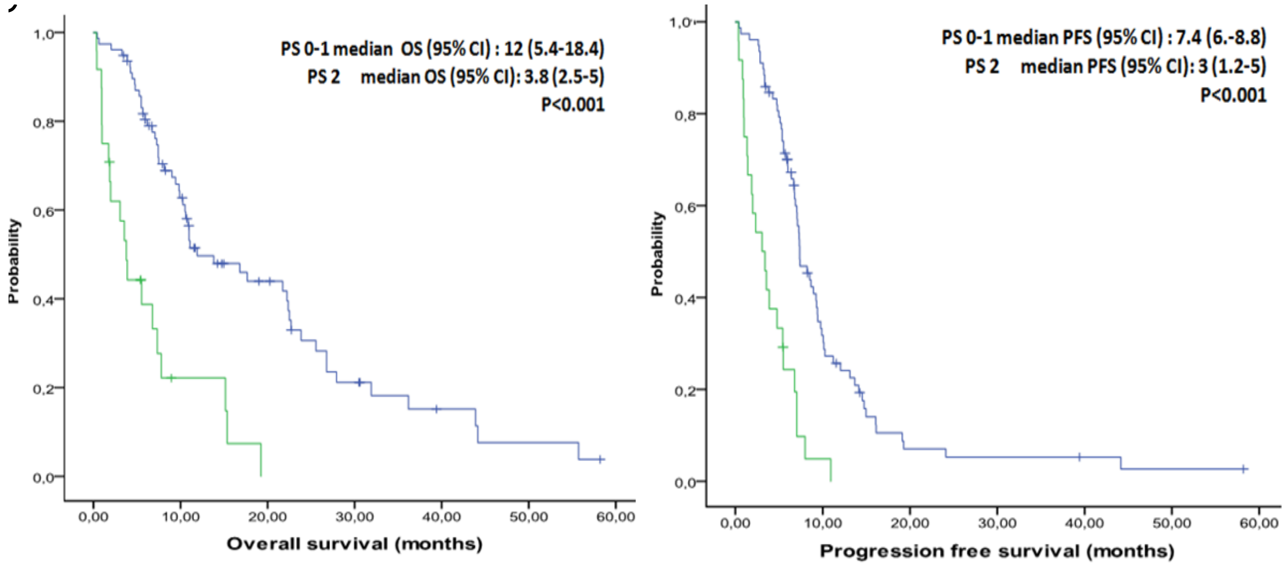

Figure S1: Kaplan-Meier curves showing overall survival (OS) and progression-free survival (PFS) for all patients according to performance status (PS)

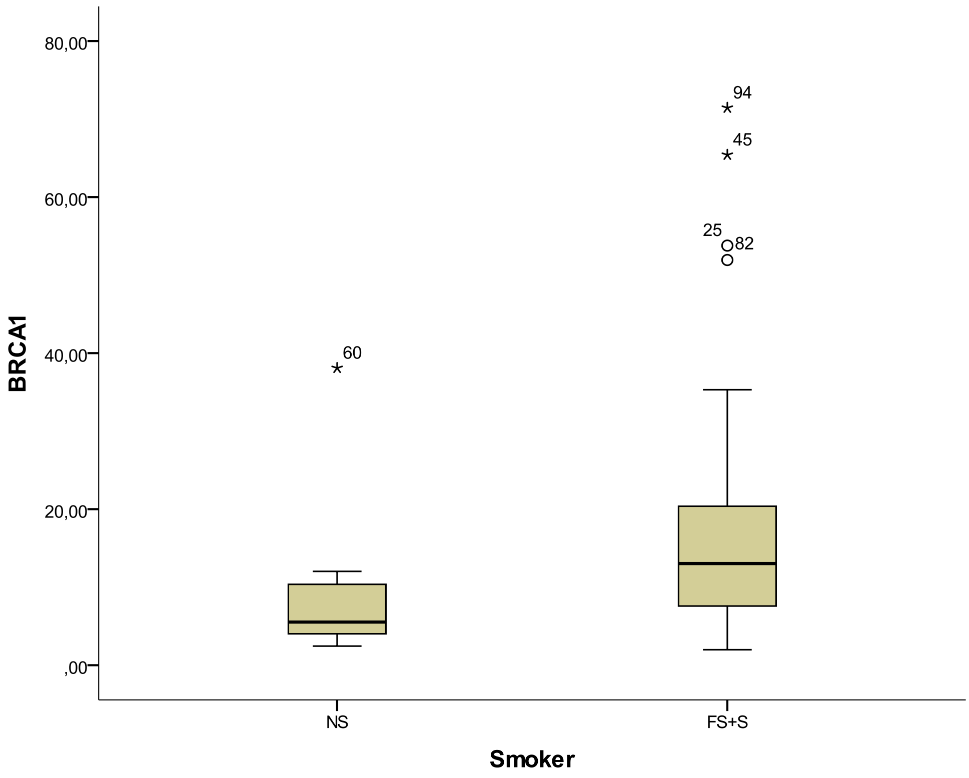

	Median (range)	P (*)
NON-SMOKERS	5.5 (2.4-38.1)	0.04
FORMER / CURRENT SMOKERS	13 (1.9-71.4)	

(*) U Mann-Whitney

Figure S2: Differential expression of BRCA1 mRNA according to smoking status. (NS: non-smokers; FS: former smokers; S: current smokers)
