# Supplementary material for: The predictive value of 53BP1 and BRCA1 mRNA expression in advanced non-small-cell lung cancer patients treated with first-line platinum-based chemotherapy
Source: Oncotarget. 2013 Jul 31;4(10):1572–81. doi: 10.18632/oncotarget.1157 (PMC3858546; doi:10.18632/oncotarget.1157)
Supplement: Supplementary file 2 [file oncotarget-04-1572-s002.pdf]

The predictive value of 53BP1 and BRCA1 mRNA expression in advanced non-small-cell lung cancer patients treated with first-line platinum-based chemotherapy - Bonanno et al

Table S1. Primers and probes used for all the analyses

Gene		Primers	Probes
**β-ACTIN**	**F**	5´ TGAGCGCGGCTACAGCTT 3´	6FAM 5´ ACCACCACGGCCGAGCGG 3´ TAMRA
	**R**	5´ TCCTTAATGTCACGCACGATTT 3´	
**BRCA1**	**F**	5´GGCTATCCTCTCAGAGTGACATTTTA 3´	6FAM 5´CCACTCAGCAGAGGG 3´ MGB
	**R**	5´GCTTTATCAGGTTATGTTGCATGGT 3´	
**MDC1**	**F**	5´ GCCCTTCCCATTGCAGCT 3´	6FAM 5´ ACAACAGGTACCCTAGATGA 3´ MGB
	**R**	5´ GGCTGTGTAGCCAGGACCTC 3´	
**UBC13**	**F**	5´ ACTTCCACTCGTGCGTGAGG 3´	6FAM 5´ CGAGAGGAGCCGGAGA 3´ MGB
	**R**	5´ GCCATCTTGTCAGAACCCGA 3´	
**RNF8**	**F**	5´ GGTGCGAGGTGACTGTAGGAC 3´	6FAM 5´AGGATTTGGTGTCACATAC 3´ MGB
	**R**	5´ GGGCAGATTTTTGATACCAGTTG 3´	
**CASPASE 3**	**F**	5´ TTTCATAAAAGCACTGGAATGACATC 3´	6FAM 5´ TGGTACAGATGTCGATGCA 3´ MGB
	**R**	5´ TCTGAATGTTTCCCTGAGGTTTG 3´	
**53BP1**	**F**	5´ GTCAGGTCATTGAGCAGTTACCTC 3´	6FAM 5´ GGACAAGCAGTGTTCT 3´ MGB
	**R**	5´ TCCTCCACAGCAGGAGCAG 3´	
**PIAS4**	**F**	5´ AGACCATTGGGGTAAAGCACC 3´	6FAM 5´ AAGGCACTGGTCAAGG 3´ MGB
	**R**	5´ GGATCAAGGCGCAGCTTCT 3´	
**UBC9**	**F**	5´ GAACTGGGAGTGCGCCAT 3´	6FAM 5´ AAGGGACTCCGTGGGA 3´ MGB
	**R**	5´ TCATCTTTGAAAAGCATCCGTAGT 3´	
**MMSET**	**F**	5´ TGTCGAAGCAGCTCTTGTGTCT 3´	6FAM 5´ CTTCAGATAAAAAGATTCCAG 3´ MGB
	**R**	5´ TCATCTTTGAAAAGCATCCGTAGT 3´	

Table S2. Number of cases in which mRNA expression was successfully analyzed and the median and tertiles cutoff values of expression levels.

SAMPLES	MDC1	MMSET	53BP1	CASP3	RNF8	UBC13	PIAS4	UBC9	BRCA1
**N**	67	75	74	64	76	82	59	80	67
**Median cutoff**	3.6	4	5.9	2.9	8.2	4.2	2.6	2.3	13
**Low tertile cutoff**	1.9	2.4	4.2	2	5.5	3.1	1.7	1.4	7.6
**High tertile cutoff**	5.4	5.7	9.6	3.7	11.2	6.4	3.5	3	17.7

Table S3. Correlations among the mRNA expression levels of the nine genes analyzed.

	MDC1	MMSET	53BP1	CASP3	RNF8	Ubc13	PIAS4	Ubc9	BRCA1
**MDC1**	1	0.38<br>P=0.003	0.44<br>P<0.001	0.32<br>P=0.02	0.59<br>P<0.001	0.62<br>P<0.001	0.50<br>P<0.001	0.50<br>P<0.001	0.47<br>P<0.001
**MMSET**		1	0.44<br>P<0.001	0.35<br>P=0.005	0.51<br>P<0.001	0.55<br>P<0.001	0.59<br>P<0.001	0.48<br>P<0.001	0.57<br>P<0.001
**53BP1**			1	0.32<br>P=0.01	0.41<br>P<0.001	0.53<br>P<0.001	0.67<br>P<0.001	0.60<br>P<0.001	0.37<br>P=0.003
**CASP3**				1	0.55<br>P<0.001	0.47<br>P<0.001	0.50<br>P<0.001	0.41<br>P=0.01	0.31<br>P=0.02
**RNF8**					1	0.57<br>P<0.001	0.64<br>P<0.001	0.38<br>P=0.001	0.39<br>P=0.01
**UBC13**						1	0.69<br>P<0.001	0.44<br>P<0.001	0.36<br>P=0.01
**PIAS4**							1	0.41<br>P=0.01	0.54<br>P=0.001
**UBC9**								1	0.09<br>P=0.55
**BRCA1**									1
